# Attitudes and behaviors of pediatric surgical nurses toward pediatric patients with obesity in China

**DOI:** 10.3389/fpubh.2026.1776677

**Published:** 2026-03-11

**Authors:** Lijun Chang, Yanhua Gao, Yakun Liu

**Affiliations:** 1 General Surgery Department, Children’s Hospital of Soochow University, Suzhou, Jiangsu, China; 2 Department of Nursing, Qilu Hospital of Shandong University, Jinan, Shandong, China

**Keywords:** attitudes toward obesity, behavioral intentions, childhood obesity, nursing education, pediatric surgical nurses, weight bias

## Abstract

**Background:**

Childhood obesity is a growing global public health issue, with increasing prevalence worldwide, including in China. The rise in obesity-related pediatric conditions requiring surgical intervention underscores the need to address this challenge in pediatric surgical care. However, research on this topic is limited. This study aimed to assess pediatric surgical nurses’ knowledge, attitudes, behaviors, and factors influencing their behavioral intentions.

**Methods:**

This is a dual-center, cross-sectional study. Nurses in direct clinical care roles at two medical institutions in China were randomly selected and completed a questionnaire assessing their knowledge, attitudes, and behavioral intentions. Factors associated with their attitudes, and behavioral intentions were investigated.

**Results:**

A total of 178 nurses participated in the study. Most demonstrated an acceptable level of obesity-related health knowledge and generally positive attitudes toward pediatric patients with obesity. However, gaps in professional preparedness and persistent weight bias were identified. Behavioral intentions were positively correlated with nurses’ BMI (*r* = 0.16, *p* = 0.04) and positive attitudes (*r* = 0.20, *p* = 0.01), and negatively correlated with perceived weight bias (*r* = −0.39, *p* < 0.01) and negative attitudes toward treating pediatric patients with obesity (*r* = −0.45, *p* < 0.01). Behavioral intentions were not correlated with obesity-related knowledge or other participant characteristics.

**Conclusion:**

Nurses’ attitudes and personal factors, rather than knowledge alone, are more correlated with caregiving behavioral intentions toward pediatric patients with obesity. Addressing biases and enhancing professional preparedness through targeted education are crucial for improving care in pediatric surgical settings in China.

## 1 Introduction

Childhood obesity is now recognized as one of the most pressing global public health issues of the 21st century (1–3). The prevalence of overweight and obesity among children has increased rapidly over recent decades, affecting both high-income and low- and middle-income countries (3). Excess body weight in childhood is associated with a wide range of adverse health outcomes, including metabolic disorders, cardiovascular risk factors, musculoskeletal problems, and psychological distress (4, 5). Moreover, childhood obesity often persists into adulthood, substantially increasing the lifetime risk of chronic diseases and premature mortality (4). Beyond its health consequences, childhood obesity also imposes a considerable socioeconomic burden on both families and healthcare systems (6).

The burden of childhood obesity is increasing particularly rapidly in China (7). It has been projected that by 2030, the prevalence of obesity among Chinese children and adolescents will reach approximately 15.1% (7). In parallel with this rising prevalence, pediatric surgical care constitutes a substantial component of health systems worldwide. Recent global estimates indicate that an additional 54 million pediatric surgical procedures are needed annually to meet the unmet demand for children’s surgical care (8). Within this broader surgical landscape, obesity-related pediatric conditions requiring operative management are becoming increasingly common. Childhood obesity is significantly associated with a variety of surgical diseases, such as slipped capital femoral epiphysis, gallstone disease, and other obesity-related complications. In addition, obesity has been linked to increased perioperative risks across a broad range of pediatric procedures, including anesthetic challenges, respiratory complications, and prolonged hospital stay (4, 5, 9, 10). Previous research on weight bias in healthcare has primarily focused on physicians, medical trainees, or mixed professional groups (11, 12). Although these studies provide important insights into healthcare professionals’ perceptions of obesity, relatively limited attention has been given specifically to nurses, particularly within pediatric surgical settings. Pediatric surgical nurses play a critical role in the perioperative care of these children, including clinical monitoring, technical nursing procedures, patient education, and psychosocial support (10). However, caring for children with obesity may involve greater physical workload, increased technical difficulty, and heightened safety concerns, all of which may influence nurses’ attitudes and their potential caregiving behaviors. Personal positive or negative attitudes reflect individual evaluations that may directly shape caregiving intentions (13). Perceived weight bias among colleagues represents the broader clinical climate and social norms and may also influence intended behaviors (14). Both attitudes and perceived professional norms have been suggested to affect behavioral intentions through different but related pathways (13, 14). Importantly, nurses’ attitudes and potential behaviors are closely linked to the quality of nursing care, patient experiences, and clinical outcomes (15). Hence, unrecognized negative attitudes may represent a modifiable but often overlooked risk factor in pediatric surgical care.

Despite the rising prevalence of childhood obesity and the increasing number of pediatric surgical patients with obesity, evidence on pediatric surgical nurses’ attitudes and potential behaviors remains limited, particularly in China. Most studies focus on adult or general nursing settings, making it increasingly urgent to understand attitudes, behaviors, and their influencing factors in pediatric surgical contexts (16, 17). Therefore, the present study conducted a dual-center, questionnaire-based cross-sectional survey to examine pediatric surgical nurses’ attitudes toward children with obesity, their potential caregiving behaviors, and associated influencing factors. We aim to provide empirical evidence to optimize clinical nursing practice, inform targeted education and management strategies, and support healthcare systems in addressing the continuing rise in childhood obesity.

## 2 Materials and methods

### 2.1 Study design and setting

This was a questionnaire-based cross-sectional study conducted in two medical institutions in China. One center was a large tertiary general hospital serving as a regional medical center for approximately 90 million people in Shandong Province. The other center was a municipal children’s hospital providing specialized pediatric services in city with a population of approximately 12 million in Jiangsu Province. The study protocol was reviewed and approved by the institutional ethics committees. All participants were informed of the study objectives and procedures before participation and provided electronic informed consent. The study was conducted in accordance with the principles of the Declaration of Helsinki and its amendments.

### 2.2 Participants

Eligible participants were registered nurses or nursing professionals working in direct clinical care positions within hospital departments that routinely provide surgical care or perioperative nursing for pediatric patients. These departments included, but were not limited to, pediatric general surgery, pediatric orthopedics, surgical units in general hospital providing pediatric services (e.g., cardiothoracic surgery), operating rooms, and post-anesthesia care units. Participants were required to have direct clinical contact with pediatric patients and to be able to complete the questionnaire independently.

Exclusion criteria were as follows: (1) refusal to participate or failure to provide informed consent; (2) self-reported average care of fewer than one child with obesity per month; (3) non-clinical positions (e.g., administrative roles) or trainees without independent clinical responsibilities; (4) incomplete questionnaires or withdrawal before completion; (5) nurses working exclusively in specialized bariatric or metabolic surgery teams; (6) low-quality questionnaires, defined *a priori* as those with an abnormally short completion time (less than a predefined minimum threshold based on the number of items), obvious response patterns (e.g., identical responses to nearly all items), or logically inconsistent anthropometric data.

### 2.3 Sampling and data collection

Participants were selected using a stratified random sampling approach. In each institution, eligible nurses were identified from departmental rosters and assigned unique study codes. A computer-generated random number list was used to select participants proportionally from each department. After providing informed consent, participants received access to the electronic questionnaire via a web link or QR code, which could be completed anonymously using a mobile device or computer. Each participant was allowed to submit the questionnaire only once.

### 2.4 Measures

The questionnaire collected information on sex, age, years of clinical practice, academic qualifications, and current department. Age was collected as a categorical variable using predefined age groups. Self-reported height and weight were used to calculate body mass index (BMI, kg/m^2^). According to World Health Organization criteria, overweight was defined as BMI ≥ 25 and <30 kg/m^2^, and obesity as BMI ≥ 30 kg/m^2^. Overweight or obesity was defined as BMI ≥ 25. Participants were also asked whether they had family members with obesity.

Knowledge of obesity-related health risks was assessed using the Obesity Risk Knowledge Scale (ORK-10) (18). The ORK-10 consists of 10 items assessing knowledge of medical risks associated with obesity. Each item is scored as correct (1 point) or incorrect/do not know (0 points), yielding a total score ranging from 0 to 10. Higher scores indicate better knowledge of obesity-related health risks. A score of 0–2 points indicates very poor knowledge; 3–5 points indicates poor knowledge; 6–7 points indicates good knowledge; and 8–10 points indicates very good knowledge. The ORK-10 scale has been reported to be reliable, discriminant and valid, with a Cronbach alpha of >0.8 (19).

Nurses’ attitudes toward children with obesity were assessed using the Attitudes of Health Care Providers about Treating Patients with Obesity scale (20). This instrument is designed to evaluate explicit weight-related attitudes and perceptions among health care providers and has been widely used in clinical and research settings. Previous studies have demonstrated good internal consistency of this scale, with reported Cronbach’s alpha coefficients ranging from 0.73 to 0.90 (21, 22). The scale consists of 22 items and comprises three subscales, including positive attitudes toward treating patients with obesity (4 items), negative attitudes toward treating patients with obesity (14 items), and perceived weight bias among other health care professionals (4 items). Each item is rated on a 5-point Likert scale, ranging from 1 (“strongly disagree”) to 5 (“strongly agree”). Subscale scores are calculated by averaging item responses within each subscale, with higher scores indicating stronger endorsement of the corresponding attitudes. Percent agreement for each item was also calculated by summing the frequency of “agree” and “strongly agree.”

Behavioral intentions were assessed using four clinical vignettes developed by senior nursing professionals experienced in pediatric surgery and perioperative care. The vignette-based approach was used to evaluate intended nursing behaviors in realistic, time-pressured clinical situations while minimizing social desirability bias. The vignettes reflected common pediatric surgical or perioperative scenarios involving children with obesity, including postoperative monitoring, emergency intravenous access, postoperative pain management, and preoperative preparation for urgent surgery. The vignettes were developed through a structured process. First, common perioperative scenarios involving children with obesity were identified based on clinical experience and relevant literature (23). Draft vignettes were reviewed by a panel of three senior pediatric surgical nurses and one pediatric surgeon to ensure clinical relevance and content validity. The revised vignettes were pilot-tested among five nurses who were not included in the final sample to assess comprehensibility and completion time. Each vignette included two response options: one reflecting immediate assistance and the other reflecting delayed or conditional assistance. Participants rated each option on a 7-point scale (1 = very unlikely to 7 = very likely). Scores for the delayed/conditional option were reverse-coded. For each vignette, the two option scores were averaged. The overall behavioral intention score was calculated as the mean of the four vignette scores, with higher values indicating stronger intention to provide timely care.

### 2.5 Data quality control

Several measures were implemented to ensure data quality. The questionnaire was pre-tested by members of the research team, yielding an average completion time of 5.3 min. Based on this pre-test, questionnaires completed in less than 2.5 min were considered potentially inattentive and were excluded. Logical consistency checks were performed for key variables, including identification of uniform response patterns (e.g., identical responses across most items) and verification of plausible ranges for height and weight. Questionnaires with excessive missing data or implausible anthropometric values were excluded from analysis.

### 2.6 Statistical analysis

Statistical analyses were conducted using SPSS 24.0 (IBM Corp. Armonk, NY, USA). Continuous variables were examined for normality using graphical methods and formal tests. Normally distributed variables were summarized as means and standard deviations (SDs), while non-normally distributed variables were summarized as medians and interquartile ranges (IQRs). Categorical variables were presented as frequencies and percentages. Internal consistency of the nurses’ attitudes and the behavioral intention measure was assessed using Cronbach’s alpha. Correlations between obesity-related knowledge (ORK-10 scores), attitudes toward obesity scale, behavioral intention scores, and participant characteristics (including BMI) were examined using Pearson or Spearman correlation coefficients, as appropriate. Multivariable linear regression analyses were conducted to examine the independent associations between knowledge, attitudes, and behavioral intention. The models were adjusted for demographic covariates, including sex, age group, years of clinical practice, BMI, academic qualification, and family history of obesity. Sensitivity analyses were conducted by institution to assess the robustness of the findings. All statistical tests were two-sided, and a *p* value <0.05 was considered statistically significant.

## 3 Results

A total of 200 eligible participants were randomly selected and invited to participate in the study. Eleven nurses (5.5%) declined participation. Of the 189 questionnaires returned (response rate: 94.5%), 11 (5.8%) were excluded after data quality control due to excessively short completion time or clear response patterns indicative of inattentive responding (*n* = 9) and logical inconsistencies in key variables (*n* = 2). Consequently, data from 178 participants (89.0%) were included in the final analysis.

Among the included participants, 98 (55.1%) were from outpatient clinics, emergency departments, or operating rooms, and 80 (44.9%) from inpatient wards. The sample comprised 147 females (82.6%) and 31 males (17.4%). The demographic and professional characteristics of the participants are summarized in Table 1.

**Table 1:** Characteristics of participants.

Characteristic	Participants (*n* = 178)
Sex	
Female	147 (82.6%)
Male	31 (17.4%)
BMI (kg/m^2^), mean (SD)	22.8 (3.6)
Overweight or obesity	
Yes	39 (21.9%)
No	139 (78.1%)
Age group	
≤25	26 (14.6%)
26–30	39 (21.9%)
31–35	48 (27.0%)
36–40	50 (29.1%)
≥41	15 (8.4%)
Years of clinical practice (years), mean (SD)	10.9 (6.8)
Academic qualification	
Associate degree	19 (10.7%)
Bachelor’s degree	154 (86.5%)
Postgraduate	5 (2.8%)
Family member with obesity	
Yes	76 (42.7%)
No	102 (57.3%)

BMI, body mass index; SD, standard deviation.

### 3.1 Obesity-related knowledge

The mean ORK-10 score among participants was 6.3 (1.5), indicating an overall acceptable level of obesity-related knowledge. The ORK-10 demonstrated acceptable internal consistency in the present sample (Cronbach’s *α* = 0.73). Based on predefined score categories, 95 (53.3%) participants demonstrated good knowledge and 33 (18.5%) demonstrated very good knowledge of obesity-related health risks.

Years of clinical experience were weakly but significantly negatively correlated with ORK-10 scores (*r* = −0.16, *p* = 0.04). No significant associations were observed between ORK-10 scores and BMI (*r* = 0.10, *p* = 0.19), sex, department type, family history of obesity, academic qualification, or age.

### 3.2 Attitudes toward treating children with obesity

The mean score for the subscale reflecting positive attitudes toward treating patients with obesity was 4.1 (0.8), with 46 participants (25.8%) scoring the maximum value of 5, indicating strongly positive attitudes. The median scores for the negative attitudes subscale and the perceived weight bias among other professionals subscale were 2.1 (0.8) and 2.1 (1.0), respectively, suggesting generally low levels of negative attitudes and perceived professional bias. Overall, 77.5% of participants agreed that children with obesity should be treated with compassion and respect. However, only 69.1% reported feeling professionally prepared to effectively care for children with obesity. Additionally, 25.3% reported having witnessed colleagues making negative comments about patients with obesity. While only 2.8% agreed with the statement that treating a patient with obesity was repulsive, as many as 29.8% agreed that children with obesity were lazy.

No significant associations were observed between positive attitude scores and sex, age, academic qualification, overweight or obesity status, or family history of obesity. Positive attitude scores were not significantly correlated with obesity-related knowledge (*r* = 0.12, *p* = 0.11), BMI (*r* = 0.06, *p* = 0.45), or years of clinical experience (*r* = −0.05, *p* = 0.55).

Similarly, negative attitude scores were not significantly associated with BMI (*r* = 0.08, *p* = 0.30), obesity-related knowledge (*r* = −0.03, *p* = 0.70), years of clinical experience (*r* = −0.10, *p* = 0.19), or other demographic and professional variables. Scores reflecting perceived weight bias among other professionals were also not significantly correlated with BMI (*r* = 0.01, *p* = 0.87), obesity-related knowledge (*r* = −0.01, *p* = 0.91), or years of experience (*r* = −0.12, *p* = 0.12).

### 3.3 Behavioral intentions toward children with obesity

The behavioral intention measure demonstrated good internal consistency (Cronbach’s *α* = 0.83). The mean score reflecting willingness to provide timely and proactive care to children with obesity was 6.1 (1.1), indicating a generally high level of positive behavioral intention.

Behavioral intention scores were positively correlated with BMI (*r* = 0.16, *p* = 0.04), suggesting that nurses with higher BMI were more likely to report immediate willingness to assist children with obesity. Nurses who were overweight or obese demonstrated significantly higher behavioral intention scores compared with those who were not overweight or obese (6.4 (0.8) vs. 5.9 (1.1), *p* = 0.03). No significant differences in behavioral intention scores were observed according to other demographic and professional variables.

Behavioral intention scores were significantly positively correlated with positive attitudes toward treating patients with obesity (*r* = 0.20, *p* = 0.01) and significantly negatively correlated with perceived weight bias among other professionals (*r* = −0.39, *p* < 0.01) and negative attitudes toward treating patients with obesity (*r* = −0.45, *p* < 0.01). Correlations among key study variables are presented in Table 2.

**Table 2:** Correlations between obesity knowledge, attitudes, and behavioral intention.

Variables	1	2	3	4	5
1. Knowledge	–	0.12	0.01	−0.03	−0.01
2. Positive attitudes	0.12	–	−0.07	−0.10	0.20^*^
3. Perceptions of weight bias	0.01	−0.07	–	0.64^*^	−0.39^*^
4. Negative attitudes	−0.03	−0.10	0.64^*^	–	−0.45^*^
5. Behavioral intention	−0.01	0.20^*^	−0.39^*^	−0.45^*^	–

Pearson correlation coefficients are presented.

* *p* < 0.05.

After adjustment for demographic covariates, multivariable linear regression analyses confirmed that positive attitudes, perceived weight bias, and negative attitudes were significantly associated with behavioral intention (Table 3). The models explained between 5 and 21% of the variance in behavioral intention, with negative attitudes showing the strongest explanatory power (adjusted *R*^2^ = 0.21).

**Table 3:** Multivariable linear regression analysis of factors associated with behavioral intention.

Predictor	*B* (95% CI)	Standardized *β*	*p* value	Adjusted *R*^2^	*F*-statistic	Model *p* value
Knowledge	−0.03 (−0.15 to 0.08)	−0.05	0.55	0.01	1.30	0.25
Positive attitudes	0.25 (0.06–0.43)	0.19	0.01	0.05	2.24	0.03
Perceptions of weight bias	−0.42 (−0.56 to −0.28)	−0.40	<0.01	0.17	6.19	<0.01
Negative attitudes	−0.59 (−0.75 to −0.40)	−0.45	<0.01	0.21	7.73	<0.01

Model adjusted for sex, age group, years of clinical practice, BMI, academic qualification, and family history of obesity. Separate multivariable regression models were constructed for each predictor.

### 3.4 Sensitivity analysis

Sensitivity analyses demonstrated consistent patterns of associations across the two centers, supporting the robustness of the study findings (Supplementary Table S1). Formal interaction testing between institution and key predictors was not conducted due to limited statistical power after stratification.

## 4 Discussion

In this study we found that nurses in China demonstrated an overall acceptable level of obesity-related knowledge and generally positive explicit attitudes toward children with obesity. Most participants reported a high willingness to provide timely and proactive care in vignette-based clinical scenarios. However, important gaps were identified, including limited perceived professional preparedness, the persistence of negative stereotypes, and perceived weight bias within the professional environment, despite low levels of self-reported negative attitudes. Importantly, behavioral intentions were significantly associated with nurses’ attitudes toward obesity and their own BMI, whereas obesity-related knowledge was not. These findings indicate that attitudes and personal factors, rather than knowledge alone, may be more closely related to intended caregiving behaviors toward children with obesity in pediatric surgical care. As a result, interventions aimed at improving obesity care should focus not only on enhancing knowledge but also on addressing personal biases and attitudes.

The findings of this study align with a previous study conducted in USA in which pediatric surgical nurses generally exhibited positive attitudes toward children with obesity (24). While the negative attitudes and perceived professional bias observed in our study were not uncommon, they were less frequent compared to those reported by Thompson et al. (24). For example, Thompson et al. (25, 26) reported much higher proportions of negative comments (69%) and negative stereotypes (55%) about children with obesity. Another finding was the weak association between obesity-related knowledge and behavioral intentions, which contrasts with some studies where knowledge directly correlated with care behaviors. This discrepancy may be attributed to several factors, including differences in research methodology. For example, studies using more comprehensive behavioral assessments or longitudinal designs might capture more nuanced relationships between knowledge and actions (26–28). Additionally, cultural and regional differences could play a role, as healthcare systems in China may emphasize different aspects of nurse training compared to those in Western countries (29, 30). Additionally, the varying professional backgrounds of nurses across studies may also contribute to differences in how obesity-related knowledge is applied in practice (31).

The relationship between nurses’ attitudes, weight bias, and behavioral intentions is complex and multifaceted. In this study, positive attitudes toward children with obesity were associated with higher behavioral intentions to provide proactive care, while negative weight bias was linked to lower behavioral intentions. Previous research supports this finding, demonstrating that negative weight bias, such as the belief that patients with obesity are non-compliant or difficult to manage, negatively impacts healthcare providers’ actions and willingness to engage with patients with obesity (24, 32). Robstad et al. (26) reported that implicit pro-thin stereotypes and explicit agreement with anti-obesity statements were significantly associated with willingness to assist overweight patients immediately in clinical scenarios. These findings highlight the importance of addressing negative weight bias in nursing education and fostering a supportive, non-judgmental environment to enhance the quality of care provided to patients with obesity (31).

The interaction between weight bias and personal factors, such as nurses’ own BMI, can also influence caregiving behavior. Nurses with higher BMI may show more empathy and understanding, which aligns with their increased willingness to assist pediatric patients with obesity (33). Another possible explanation is that nurses with higher BMI may feel more confident and less pressured when assisting pediatric patients with obesity, as they might be physically stronger than those with lower BMI.

The sources of weight bias in clinical settings are likely multifactorial. Institutional culture, heavy workload, limited structured training on obesity care, and broader societal stereotypes regarding body weight may all contribute to the presence of biased perceptions (13, 14, 34). In surgical environments, time pressure and technical demands may further reinforce task-oriented approaches that unintentionally marginalize patients with obesity (24, 35). Although the present study was not designed to identify the origins of such bias, understanding these contextual influences is important for developing effective interventions. Future research incorporating structured interviews would provide deeper insights into how institutional, professional, and societal factors shape nurses’ perceptions and behaviors toward pediatric patients with obesity.

Since obesity is significantly associated with various surgical complications, improving perioperative care for children with obesity becomes even more critical (36, 37). Improving care for children with obesity requires a multi-level approach that includes both education and organizational support (31, 38). Several educational initiatives have been developed to enhance health professionals’ competence in obesity care, including structured pediatric obesity counseling frameworks and weight-bias–reduction training programs aimed at improving communication and reducing stigma in clinical practice (39). Training programs for nurses should focus not only on enhancing knowledge about obesity-related health risks but also on addressing underlying biases and improving communication skills to foster a non-judgmental care environment. In China, capacity-building efforts and continuing education programs related to obesity management have also been gradually introduced, although training specifically tailored to pediatric surgical settings remains limited (40). Evidence suggests that when nurses feel more professionally prepared, their confidence in providing care increases, which positively impacts patient outcomes (31, 41). Additionally, creating supportive work environments that reduce time pressures and enhance team collaboration is crucial. Multidisciplinary interventions can ensure that nurses receive continuous education and are better equipped to manage the complex needs of obese pediatric patients (25).

This study has several limitations. First, the cross-sectional design of the study limits our ability to infer causality. As a result, while associations between attitudes, knowledge, and behavioral intentions were observed, the direction and nature of these relationships needs further investigation. Second, the participating institutions were large, well-resourced hospitals with relatively structured training systems and specialized pediatric surgical services. Nurses working in smaller hospitals, rural areas, or less specialized settings may face different resource constraints, training opportunities, and organizational cultures. These could influence attitudes and behavioral intentions toward children with obesity. Therefore, caution is needed when generalizing these findings to other healthcare contexts within China. Third, this study assessed behavioral intentions using vignette-based scenarios rather than observing actual clinical behaviors. Although behavioral intention is generally considered a proximal predictor of behavior, it does not necessarily translate into real-world actions, particularly in complex and time-constrained surgical environments. Factors such as workload, institutional policies, team dynamics, and situational constraints may influence whether intended actions are enacted in practice. Therefore, our findings should be interpreted as reflecting nurses’ stated intentions rather than confirmed clinical behaviors. Finally, although we controlled for some sources of bias, self-reported data may still be influenced by social desirability bias, potentially affecting the accuracy of the responses. Future studies should address these limitations by using longitudinal designs, larger and more diverse samples, and objective measures of behavior. This study has strengths. It focuses on pediatric surgical nurses, an underexplored population in obesity-related research, and uses validated instruments with multivariable analyses to enhance methodological rigor.

## 5 Conclusion

This study emphasizes the crucial role of nurses’ attitudes and personal factors in influencing their care behaviors toward pediatric patients with obesity. Although pediatric surgical nurses demonstrated adequate knowledge and generally positive attitudes, gaps in professional preparedness and the persistence of weight bias were observed. These findings highlight the importance of addressing both biases and professional development through targeted training programs. Such efforts are essential for improving the quality of care in pediatric surgical settings and enhancing outcomes for pediatric patients with obesity.

## Data Availability

The raw data supporting the conclusions of this article will be made available by the authors without undue reservation.
