# Supplementary material for: Attitudes and behaviors of pediatric surgical nurses toward pediatric patients with obesity in China
Source: Front Public Health. 2026 Mar 11;14:1776677. doi: 10.3389/fpubh.2026.1776677 (PMC13013430; doi:10.3389/fpubh.2026.1776677)
Supplement: Supplementary file 1 [file Table_1.DOCX]

**Table S1**.Sensitivity analysis of factors associated with behavioral intention by institution.

|  | Institution A (n=98) | | | | | | Institution B (n=80) | | | | | |
| --- | --- | --- | --- | --- | --- | --- | --- | --- | --- | --- | --- | --- |
|  | B (95% CI) | Standardized β | *P* value | Adjusted R² | F-statistic | Model *P* value | B (95% CI) | Standardized β | *P* value | Adjusted R² | F-statistic | Model *P* value |
| Knowledge | 0.05 (-0.10 to 0.20) | 0.07 | 0.52 | 0.02 | 1.23 | 0.29 | -0.09 (-0.28 to 0.10) | -0.11 | 0.37 | 0.01 | 1.16 | 0.34 |
| Positive attitudes | 0.33 (0.05 to 0.61) | 0.23 | 0.02 | 0.07 | 2.02 | 0.06 | 0.19 (-0.09 to 0.46) | 0.15 | 0.18 | 0.03 | 1.31 | 0.26 |
| Perceptions of weight bias | -0.41 (-0.58 to -0.24) | -0.43 | <0.01 | 0.21 | 3.68 | <0.01 | -0.43 (-0.69 to -0.18) | -0.37 | <0.01 | 0.14 | 2.81 | 0.01 |
| Negative attitudes | -0.60 (-0.83 to -0.38) | -0.48 | <0.01 | 0.24 | 5.47 | <0.01 | -0.43 (-0.80 to -0.26) | -0.40 | <0.01 | 0.17 | 3.68 | <0.01 |

Model adjusted for sex, age group, years of clinical practice, BMI, academic qualification, and family history of obesity. Separate multivariable regression models were constructed for each predictor.
